# VP22 core domain from *Herpes simplex virus 1* reveals a surprising structural conservation in both the *Alpha*- and *Gammaherpesvirinae* subfamilies

**DOI:** 10.1099/vir.0.000078

**Published:** 2015-06

**Authors:** Kelly Hew, Sue-Li Dahlroth, Lucy Xin Pan, Tobias Cornvik, Pär Nordlund

**Affiliations:** ^1^​ Division of Structural Biology and Biochemistry, School of Biological Sciences, Nanyang Technological University, 138673, Singapore; ^2^​ Division of Biophysics, Department of Medical Biochemistry and Biophysics, Karolinska Institutet, Stockholm 171 11, Sweden

## Abstract

The viral tegument is a layer of proteins between the herpesvirus capsid and its outer envelope. According to phylogenetic studies, only a third of these proteins are conserved amongst the three subfamilies (*Alpha*-, *Beta*- and *Gammaherpesvirinae*) of the family *Herpesviridae*. Although some of these tegument proteins have been studied in more detail, the structure and function of the majority of them are still poorly characterized. VP22 from *Herpes simplex virus 1* (subfamily *Alphaherpesvirinae*) is a highly interacting tegument protein that has been associated with tegument assembly. We have determined the crystal structure of the conserved core domain of VP22, which reveals an elongated dimer with several potential protein–protein interaction regions and a peptide-binding site. The structure provides us with the structural basics to understand the numerous functional mutagenesis studies of VP22 found in the literature. It also establishes an unexpected structural homology to the tegument protein ORF52 from *Murid herpesvirus 68* (subfamily *Gammaherpesvirinae*). Homologues for both VP22 and ORF52 have been identified in their respective subfamilies. Although there is no obvious sequence overlap in the two subfamilies, this structural conservation provides compelling structural evidence for shared ancestry and functional conservation.

## Introduction

Human herpesviruses are known to cause vastly different diseases/illnesses that range from mild oral-facial blisters and chicken pox to fatal conditions such as Burkitt’s lymphoma and Kaposi’s sarcoma (Antman & Chang, 2000; Davison, 2007; Whitley & Roizman, 2001). Herpesviruses are large DNA viruses that share an overall common virion structure. The virion consists of a dsDNA encapsidated within an icosahedral capsid (Davison, 2007). Between the capsid and the outer membrane lies a layer of proteins, collectively known as the tegument (Guo *et al.*, 2010). Twenty-four different tegument proteins have been identified in *Herpes simplex virus 1* (HSV-1; *Human herpesvirus 1*) but, judging from sequence alignments, only a third of them are conserved across all the subfamilies (*Alpha*-, *Beta*- and *Gammaherpesvirinae*) of the family *Herpesviridae* (Kelly *et al.*, 2009). Tegument proteins can occur in several thousands of copies inside the virion, whilst others are less abundant (Elliott & Meredith, 1992). Some tegument proteins have been found to contribute greatly to viral entry, assembly and egress, whilst others play vital roles in viral immune evasion and regulation of viral gene expressions (Kalejta, 2008; Kelly *et al.*, 2009; Sathish *et al.*, 2012).

VP22 is a highly abundant tegument protein in HSV-1 that has been suggested, based on sequence analysis, to be unique to the alphaherpesviruses. VP22 has been suggested to be important for the secondary tegumentation of the virion and the accurate localization of several important herpesviral proteins, including the transcription activating protein VP16, the outer capsid protein VP26, the interesting E3 ubiquitin ligase ICP0, the major transcriptional regulatory protein ICP4 and the essential multifunctional ICP27 (Brignati *et al.*, 2003; Elliott & Meredith, 1992; Farnsworth *et al.*, 2007; Maringer & Elliott, 2010; Potel & Elliott, 2005; Tanaka *et al.*, 2012; Yu *et al.*, 2010). Recently, VP22 has emerged as a key node in the HSV-1 tegument–glycoprotein network, where it makes multiple protein–protein interactions and plays a selective role in the tegument acquisition of viral glycoproteins gE, gD and gM (Chi *et al.*, 2005; Elliott *et al.*, 1995; Farnsworth *et al.*, 2007; Hafezi *et al.*, 2005; Maringer *et al.*, 2012; O’Regan *et al.*, 2007a, 2010; Potel & Elliott, 2005; Stylianou *et al.*, 2009). VP22 also binds directly to cellular proteins like chromatin remodelling protein (TAF-1) and non-muscle myosin II (NMII) (van Leeuwen *et al.*, 2002, 2003). It has also been associated with interactions with cellular membranes, microtubules and nucleic acids (Brignati *et al.*, 2003; Elliott & O’Hare, 1998; Martin *et al.*, 2002; Sciortino *et al.*, 2002). Interestingly VP22 exhibits transfection potential and has been used successfully in several studies to target therapeutic DNA to specific cells of interest, such as stem cells (Bennett *et al.*, 2002; Elliott & O’Hare, 1999; Jin *et al.*, 2013; Lai *et al.*, 2000).

Sequence analysis and secondary structure predictions reveal that VP22 consists of a non-conserved N-terminal domain and a conserved C-terminal domain with the clear presence of secondary structures (O’Regan *et al.*, 2007a). Deletion and functional studies have shown that the conserved C-terminal domain in VP22 is important for binding to VP16 and gE (O’Regan *et al.*, 2007a, b). To generate insight into the VP22 structure and function, we crystallized and solved the structure of the conserved C-terminal domain of this protein, hereafter referred to as VP22_core_, to a resolution of 1.9 Å. VP22_core_ exists as a dimer with a highly conserved dimerization site. Although sequence homology of VP22 has only been established within the alphaherpesviruses, the crystal structure reveals that it shares extensive structural similarity with ORF52 from *Murid herpesvirus 68* (MHV-68) (subfamily *Gammaherpesvirinae*). ORF52_MHV-68_ has been found to be essential for replication in MHV-68 *in vitro* (Song *et al.*, 2005). Similar to VP22_core_, ORF52_MHV-68_ is also a highly expressed tegument protein that exists as a dimer made up of two identical monomers (Benach *et al.*, 2007; Bortz *et al.*, 2007). It is well conserved within the gammaherpesviruses, and has been implicated to be important for tegument association and interactions (Bortz *et al.*, 2007; Fossum *et al.*, 2009; Rozen *et al.*, 2008; Uetz *et al.*, 2006). These are coincidentally similar to some of the proposed functions of VP22 (Brignati *et al.*, 2003; Farnsworth *et al.*, 2007). With the VP22_core_ structure in hand, we have been able to compare the two protein structures, revisit the outcome of reported mutational studies as well as identify completely conserved residues that might be important for function.

## Results and Discussion

### Structure of VP22_core_

**Fig. 1.**
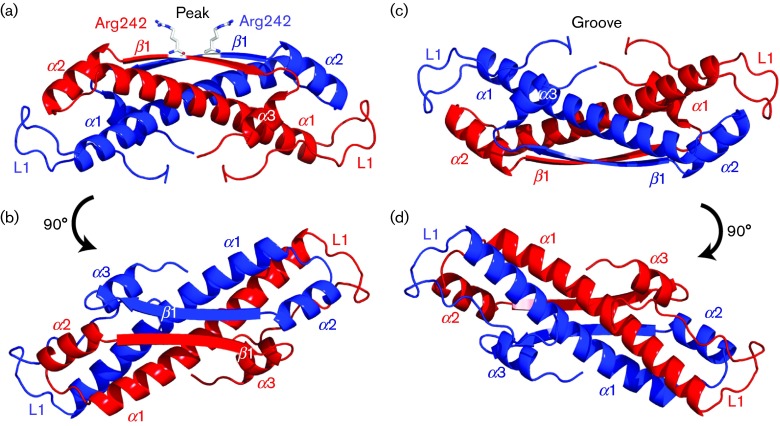
Different views of the crystal structure of the VP22_core_ dimer. (a) Each monomer (coloured red and blue) consists of three α-helices (α1–α3) and one β-strand (β1). The monomers of VP22_core_ coil around each other. The flat β1 from both monomers create a plateau with a conserved arginine (Arg242) sticking up like a peak. We refer to this face of the protein as the ‘peak side’. (b) A 90° rotation of the dimer gives the top view of the peak side. (c) The opposite side reveals a groove that is created by L1 and α1 from both monomers. (d) A 90° rotation of the dimer gives the top view of the groove side.

**Fig. 2.**
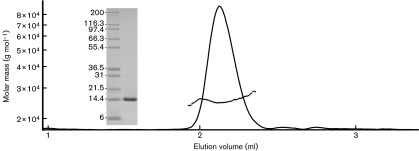
Light-scattering curve of VP22_core_ in solution as a function of its elution volume. The monomeric molar mass of VP22_core_ is 14551 g mol^−1^ and the light-scattering results show that VP22_core_ is mono-dispersed with an estimated mean molar mass of 26 500±5000 g mol^−1^. This shows that VP22_core_ is dimeric in solution. The SDS-PAGE gel of the injected VP22_core_ sample and the protein ladder (Mark12 Unstained Standard; kDa) is displayed on the left of the elution peak.

VP22_core_ crystallized in the space group P6_1_22 and the crystal structure [Protein Data Bank (PDB) ID: 4XAL] was determined at a resolution of 1.87 Å using single isomorphous replacement with anomalous scattering (SIRAS). Each asymmetrical unit consists of a molecule of VP22_core_, with visual electron density for residues 174–260, together with three amino acids from the N-terminal purification tag. The crystallographic data statistics are summarized in Table 1. The structure of VP22_core_ is constituted by a long central α-helix (α1) flanked by a long random coil (L1) at the N terminus, two shorter α-helices (α2 and α3) and one β-strand (β1) at the C terminus (Fig. 1). Two VP22_core_ monomers are related by the crystallographic twofold axis and are slightly twisted around each other, creating an elongated dimer (Fig. 1) where the α1 helices and the β1 interact in an anti-parallel fashion. The dimeric state of VP22_core_ has been proposed previously (Mouzakitis *et al.*, 2005) and our light-scattering results show that VP22_core_ is mono-dispersed with a mean molar mass of ~26 500 g mol^−1^. This is roughly twice the theoretical molecular mass of the monomeric VP22_core_ including the purification tag and the tobacco etch virus (TEV) protease site (14 551 g mol^−1^), further confirming that VP22_core_ is a dimer in solution (Fig. 2).

**Table 1.** Summary of data collection, phasing and refinement statistics

Parameter	Native (PDB ID: 4XAL)	Soaked with PbCl_2_
X-ray source	NSRRC 13C1	NSRRC 13C1
Wavelength (Å)	0.9762	0.9762
Space group	P6_1_22	P6_1_22
Unit cell parameters	*a* = 65.0, *b* = 65.0, *c* = 107.9	*a* = 65.0, *b* = 65.0, *c* = 107.6
	α = 90, β = 90, γ = 120	α = 90, β = 90, γ = 120
Resolution range (Å)	30.00–1.87 (1.94–1.87)*	27.80–1.87 (1.94–1.87)*
*I*/σ(*I*)	24.3 (3.2)*	43.8 (5.81)*
Completeness (%)	99.0 (99.6)*	100 (97.6)
Redundancy	2.9 (2.8)*	10.2 (10.2)
*R*_sym_†	0.052 (0.289)	0.047 (0.188)
Total reflections	182 439	194 305
Unique reflections	11 745	11 789
**AutoSol**		
No. of sites		1
Initial figure of merit		0.21
Figure of merit after density modification		0.63
**Refinement**		
*R*_factor_‡/*R*_free_§ (%)	20.8/25.1	
Atoms	828	
Protein residues	96	
Solvent molecules	74	
RMSD bonds (Å)	0.027	
RMSD angles (°)	0.77	
**Ramachandran quality plot**		
In preferred region (%)	99	
In allowed region (%)	1	
Outliers (%)	0	

NSRRC, National Synchrotron Radiation Research Center (Taiwan, ROC).

* Values within parentheses represent the highest resolution shell (1.939–1.872 Å).

† *R*_sym_ = 100×∑(|*I_j_*−[*I*]|)/∑(|*I*|), where the sum is calculated over all observations of a measured reflection (*I_j_*) and [*I*] is the mean intensity of all the measured observations (*I_j_*).

‡ *R*_factor_ = 100×∑(|*F*_o_|−|*F*_c_|)/∑(|*F*_o_|), where *F*_o_ and *F*_c_ are the observed and calculated structure factors, respectively.

§ *R*_free_ is equivalent to *R*_factor_, but where 5 % of the measured reflections have been excluded from refinement and set aside for cross-validation.

**Fig. 3.**
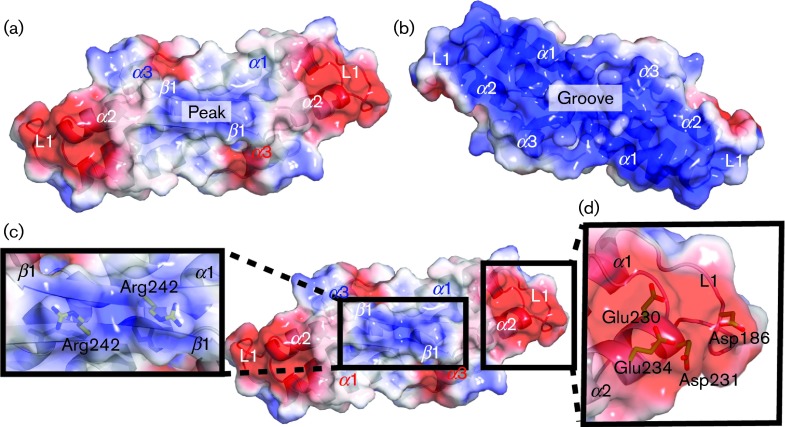
Electrostatic potential surface maps of the VP22_core_ dimer. (a) The positive, negative and uncharged regions of the surface map are coloured blue, red and white, respectively. The protein is shown in the same orientation as in Fig. 1(b). It reveals a patch of positive charges in the middle of a relatively uncharged surface. Flanking their sides are areas of negatively charged patches. (b) At the groove side, there are two large positively charged patches. The charges on this surface are contributed by the α1 amino acids lining the groove. The protein is shown in the same orientation as in Fig. 1(d). (c) The positively charged patch at the peak side is created by Arg242, whilst (d) the negatively charged patch is created by Asp186 from L1 of one monomer and a cluster of negatively charged residues, Glu230, Asp231 and Glu234, from α2 of the second monomer. These distinctively charged patches on VP22_core_ might be potential molecular interaction sites.

To be able to orientate ourselves in the structure, we have dubbed one side of the dimer the ‘peak’ (Figs 1a, b and 3a) and the other side the ‘groove’ (Figs 1c, d and 3b). On the peak side, the dimerization of β1 creates a flat plateau where two conserved arginines (Arg242) create a positively charged peak in the middle of a less charged area (Figs 1a and 3a, c). Flanking the sides of this peak are two identical negatively charged patches. The residues that contributed to these two patches are Asp186 from L1 of one VP22_core_ monomer and a cluster of negatively charged residues, Glu230, Asp231 and Glu234, from α2 of the other monomer (Figs 1c and 3a, d). The electrostatic potential surface map of the groove, which is created by L1 and α1 from both monomers, shows two large and positively charged patches. In general, distinctly charged patches on a protein surface might indicate potential sites for protein–protein interactions and any of these described areas in VP22_core_ could serve this purpose.

**Fig. 4.**
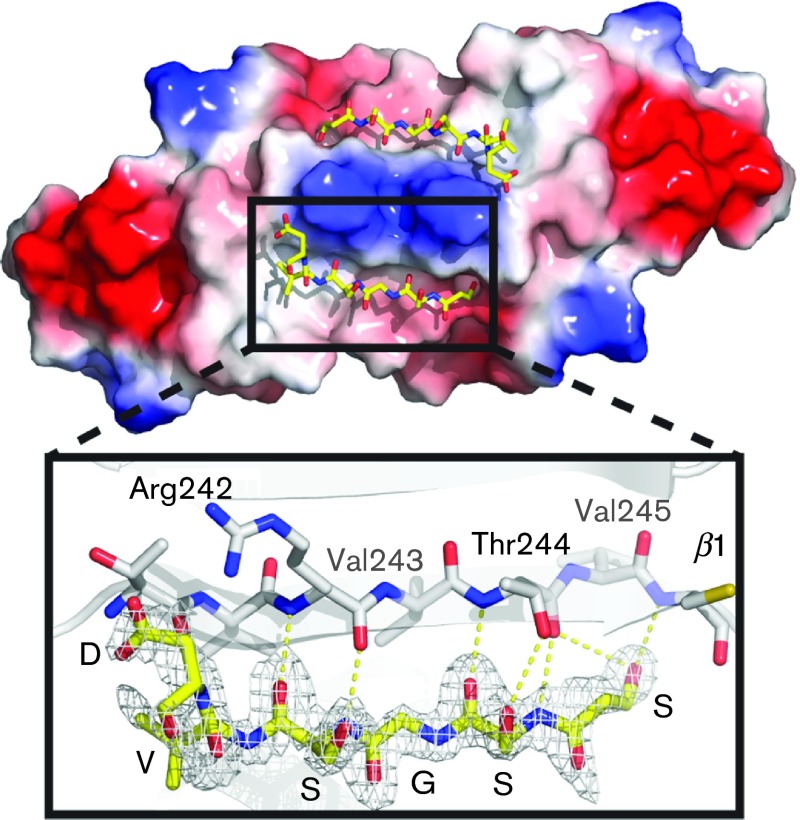
Peptide-binding site of VP22_core_ with the electrostatic potential surface map of the peak side. A peptide consisting of six amino acids was traced from the stretch of unmodelled electron density next to β1. The interaction between the peptide (yellow) and β1 (white) is magnified and displayed below. The peptide fits well into the electron density and the sequence was traced to be SSGSVD. Hydrogen bonds hold the peptide to β1 and these interactions are illustrated by yellow dotted lines.

Interestingly, we observed a stretch of unaccounted electron density next to β1 (Fig. 4). The β1 β-sheet forms a tiny β-sheet through interactions with β1 from the other monomer and contributes to the overall dimerization of VP22_core_. We managed to model a six-amino-acid peptide into this density. This peptide forms a perfect β-strand, expanding the β1 β-sheet to four stands. It corresponds to the sequence SSGSVD, which is a part of the linker region between the N-terminal His_6_-tag and the TEV protease cleavage site. This peptide is most likely contributed *in trans* from a neighbouring subunit in the crystal lattice, which is not part of the crystallographic dimer. The peptide is held tightly into place by backbone interactions and the coordination of the hydroxyl group on the N-terminal serine. Although this particular peptide sequence is most likely not of biological relevance, it indicates directly that this peptide-binding cavity could constitute a real site for protein interaction with VP22. A motif similar to the peptide was not identified at the N terminus of VP22, but it is plausible that some as-yet unidentified part of the N terminus could form a β-strand and bind in this location.

### Conserved residues in VP22_core_ contribute to its fold, oligomerization and interactions

VP22 has many proposed interaction partners. In order to evaluate and differentiate between these interactions, various deletion mutants have been created, described and discussed (Brignati *et al.*, 2003; Elliott *et al.*, 2005; Hafezi *et al.*, 2005; Martin *et al.*, 2002; O’Regan *et al.*, 2007a, b, 2010; Stylianou *et al.*, 2009) (summarized in Table S1, available in the online Supplementary Material). Whilst these studies have laid a foundation for the VP22 protein interaction network, the crystal structure of VP22_core_ can now aid in understanding these interactions at the atomic level. We mapped several of the published mutations onto the VP22_core_ structure to gain more insights into their structure–function relationship.

**Fig. 5.**
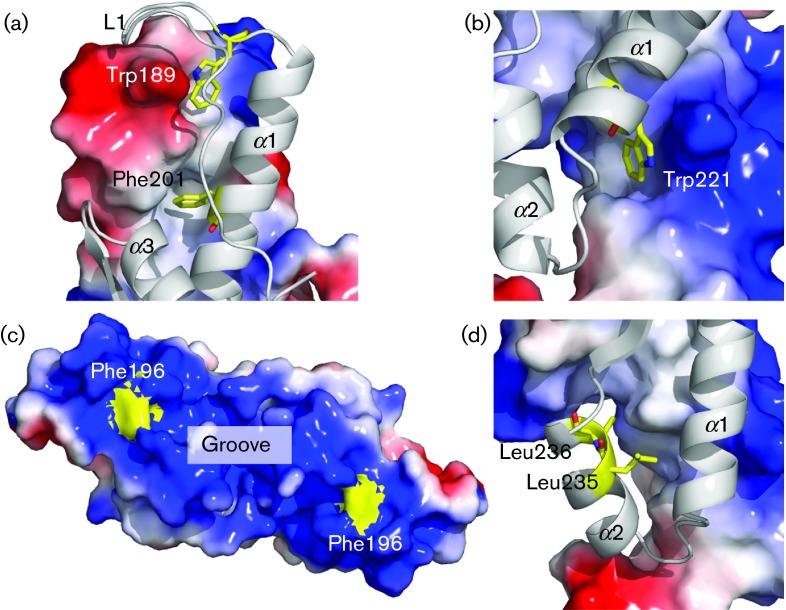
Point mutations mapped in VP22_core_. The mapped residues (a) Trp189/Phe201, (b) Trp221, (c) Phe196 and (d) Leu245/Leu246 were found to be important for VP22 protein interactions by O’Regan *et al.* (2010); (a, b, d) show one VP22_core_ monomer displayed as a cartoon and the other monomer displayed as the electrostatic potential surface map. (a) Trp189/Phe201 and (b) Trp221 are buried in the hydrophobic dimerization interface, rendering them unlikely to participate in any specific protein–protein interactions. Instead, they seem very important for dimerization. However, the surface electrostatic potential map shows that the conserved Phe196 (c) is found on the surface of the VP22_core_ and is likely to participate in protein–protein interactions. However, Leu245/Leu246 (d) are buried in the hydrophobic interface, indicating that the leucine pair is not likely to participate in specific protein–protein interactions.

Upon mapping these deletions and truncations, we can now see that most of the mutated residues that yielded in a loss of protein function are located in L1, α1 or α2 (Table S1). In most cases, the reported deletions would have removed parts of the long central helix α1 – a key secondary structure along the dimerization interface. Most of the described point mutations that seem to have an effect on VP22 interactions are also focused on this helix (O’Regan *et al.*, 2007b, 2010; Tanaka *et al.*, 2012). In particular, Trp189, Phe201 and Trp221, which have been found to disrupt the binding between VP22 and gE/VP16, are located along the dimerization interface of α1 (Fig. 5a, b). It is possible that most effects observed in these studies are the result of the distortion of VP22’s dimerization, rather than specific functional effects.

A residue of particular interest is the conserved and solvent-exposed Phe196. The electrostatic surface potential of VP22_core_ reveals that this hydrophobic Phe196 is located in the middle of the two large and highly positively charged patches at the groove side (Fig. 5c). With a single point mutation of this amino acid, O’Regan *et al.* (2010) were able to remove the binding between gE and VP22, but not between VP16 and VP22. Moreover, conserved aromatic residues on the surface of a protein have often been shown to be important for protein interactions (Albiston *et al.*, 2010; Cao *et al.*, 2008; Chouljenko *et al.*, 2012; Ferrandon *et al.*, 2003). The functional evidence from O’Regan *et al.* (2010), in combination with the high degree of conservation and strategic location/orientation of Phe196, suggest its importance in protein interactions. Given that the point mutation on Phe196 only removed the binding between gE and VP22, Phe196 and the surrounding amino acid residues may also play a key role in discriminating between the different interacting proteins of VP22 (O’Regan *et al.*, 2010).

Similarly, the binding between VP22 and VP16 was disrupted when a pair of conserved leucines along α2_VP22_ (Leu235 and Leu236) was mutated into alanines (O’Regan *et al.*, 2007b). These mutations also altered the localization sites of several HSV-1 proteins, including ICP0, gE, gD, VP16 and vhs, in the host cell (Tanaka *et al.*, 2012). However, these leucines are exposed to the hydrophobic core and do not appear to be able to participate in any direct protein–protein interactions (Fig. 5d). Thus, the loss of protein function may likely have arisen due to either the collapse of the global VP22_core_ structure or local distortions of α-helical stability/positions. If the observed effects are indeed a result of local structural distortions, these mutations highlight the significance of the entire α2 for making interactions with its binding partners.

### VP22_core_ is structurally homologous to ORF52 from MHV-68

VP22 consists of two domains where only the C-terminal domain is highly conserved in the alphaherpesviruses. The N-terminal domain is more variable and this domain is completely absent in some alphaherpesviruses (O’Regan *et al.*, 2007a). A structural homology search with the structure of VP22_core_ on the Dali server identified another herpesvirus protein, ORF52 from MHV-68 (PDB ID: 2R3H and 2OA5), with a mean *Z* score of 5.6 (Holm & Rosenström, 2010). ORF52 from MHV-68 (ORF52_MHV-68_) is a small viral protein of 21 kDa, making it substantially smaller than the full-length VP22 (35 kDa). As with VP22_core_, ORF52_MHV-68_ is also a highly expressed tegument protein that exists as a dimer made up of two identical monomers (Benach *et al.*, 2007; Bortz *et al.*, 2007). Both VP22 and ORF52_MHV-68_ are well conserved within the alpha- and gammaherpesviruses, respectively, and both proteins seem to share similar functions, such as tegument association and interactions (Bortz *et al.*, 2007; Brignati *et al.*, 2003; Fossum *et al.*, 2009; Rozen *et al.*, 2008; Uetz *et al.*, 2006). For clarity, we use the subscripts ‘VP22’ and ‘ORF52’ to differentiate between the secondary structural elements in the respective proteins.

**Fig. 6.**
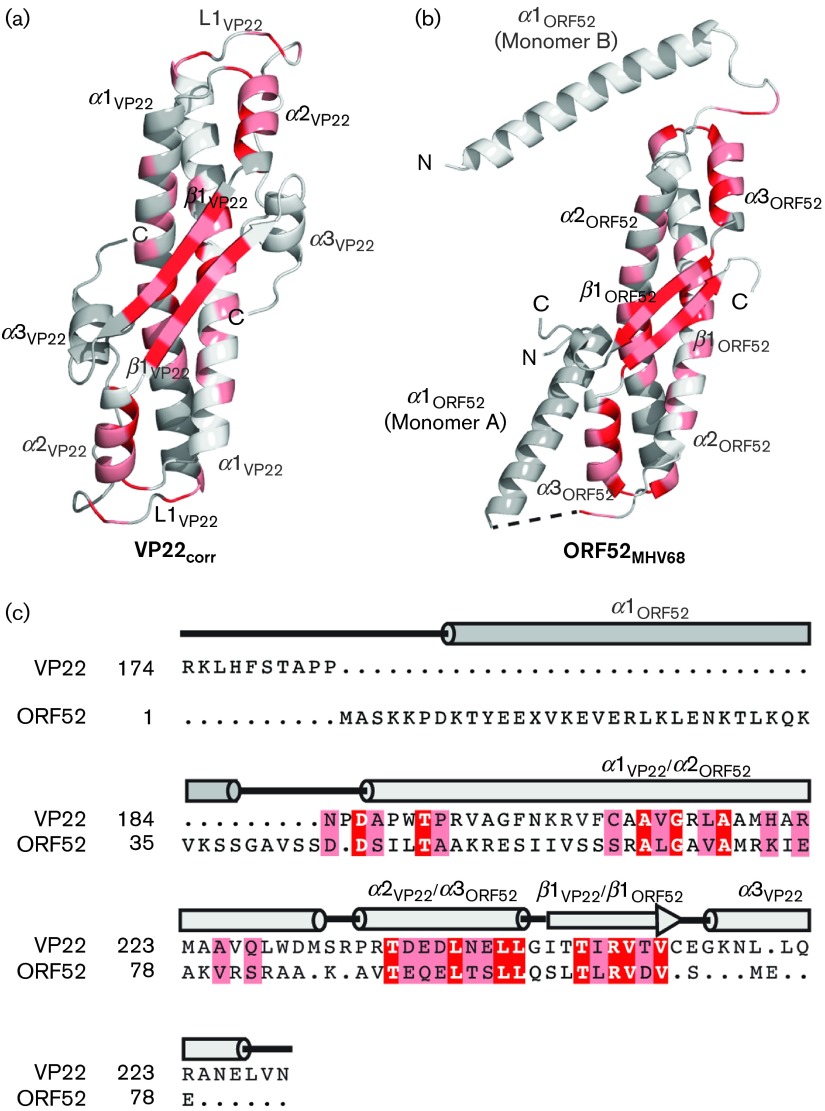
Structural and sequence alignment of VP22_core_ and ORF52_MHV-68_. The dimer structures of (a) VP22_core_ and (b) ORF52_MHV-68_ are shown as cartoons in the same orientation. (c) The structural alignment of VP22_core_ and ORF52_MHV-68_ is reproduced in a sequence alignment. The completely conserved amino acids are highlighted in red, whilst the other conserved residues are highlighted in pink. The conserved amino acids are mainly concentrated along the hydrophobic dimerization interface at α1_VP22_, α2_VP22_ and β1_VP22_.

To analyse the structural similarities in detail, the VP22_core_ structure was compared with the published dimer of ORF52_MHV-68_ (PDB ID: 2OA5) using Coot (Emsley *et al.*, 2010). The α carbons of each VP22_core_ monomer and the individual ORF52_MHV-68_ monomer align well with a mean root-mean-square deviation (RMSD) of 2.1 Å (Fig. 6a, b). Both VP22_core_ and ORF52_MHV-68_ have long central α-helices (α1_VP22_ and α2_ORF52_) that constitute the core of the dimer interactions. The anti-parallel β-strands (β1_VP22_ and β1_ORF52_) also contribute to this dimerization. The helices α2_VP22_ and α3_ORF52_ located on the surface of the proteins align well with each other. There is a slight difference at the C terminus of this superposition where we notice that whilst ORF52_MHV-68_ has an extended loop, HSV-1 VP22_core_ has an α-helix denoted α3_VP22_. However, the major differences between VP22_core_ and ORF52_MHV-68_ lie at the N terminus (Fig. 6a, b). At the N terminus, ORF52_MHV-68_ has an additional helix (α1_ORF52_), whilst VP22_core_ has a long extended loop (L1_VP22_). In ORF52_MHV-68_, this particular helix displays two different conformations by extending in different directions in the dimer structure, suggestive of a flexible N terminus in ORF52_MHV-68_. L1_VP22_ stretches in the same direction as α1_ORF52_ in chain A and in the opposite direction from α1_ORF52_ of ORF52_MHV-68_ chain B. VP22_core_ has an additional N-terminal domain, not present in our structure, and secondary structure predictions also indicate a low α-helical propensity along L1_VP22_ (not shown) (Cole *et al.*, 2008). Thus, there is a possibility that L1_VP22_ exists as a part of a long and flexible connection between VP22_core_ and its N-terminal domain.

Based on the structural similarity, we generated a structure-based sequence alignment between the monomeric VP22_core_ and ORF52_MHV-68_ yielding a sequence identity of 13 % (Fig. 6c) (Pettersen *et al.*, 2004). As with the conserved residues within the VP22 homologues, most of these residues are clustered throughout the dimerization interface and the hydrophobic core. The structure-based sequence alignment prompted us to try and identify a possible homologue in the betaherpesviruses. However, no homologue could be identified.

To further understand the sequence conservation between the alpha- and gammaherpesviruses, we generated an additional alignment with most homologues from the alpha- and gammaherpesviruses (Fig. S1). Although this sequence alignment displays very low sequence similarity, it does reveal four amino acids that are particularly conserved in both subfamilies. In particular, along α2_VP22_ and α3_MHV-68_, a leucine (Leu236_VP22_/Leu89_ORF52_) is conserved in both the alpha- and gammaherpesviruses. As in VP22, this conserved leucine in ORF52_MHV-68_ (coloured red at α3_MHV-68_ in Fig. 6b) is also exposed to the hydrophobic core of the protein, supporting the importance of oligomerization of this protein for proper function.

The remaining three amino acids that are conserved in both the alpha- and gammaherpesviruses are Arg242_VP22_/Arg95_ORF52_, Val243_VP22_/Val96_ORF52_ and Val245_VP22_/Val98_ORF52_ (Fig. S1). These amino acids are located along β1, where the side chain of the valines stretches into the core of the structure, whilst the side chain of the arginine is solvent-exposed (Figs 4 and 6). The two conserved valines seem to contribute to the fold, but the highly conserved arginine (Arg242_VP22_/Arg95_ORF52_) along β1 appears to be important for protein binding. This conserved residue is found next to our proposed peptide-binding site and is what creates the distinct peak of VP22_core_ (Figs 3a, c and 4). To underline the importance of this completely conserved arginine is the fact that Wang *et al.* (2012) could disrupt the binding between ORF52_MHV-68_ and ORF42_MHV-68_ with a single amino acid substitution (Arg→Ala) in this position. Hence, although there is no determined homologue to ORF42_MHV-68_ in HSV-1, it is likely that the corresponding mutation in VP22 could also disrupt the interaction to one or several of its (un)known binding partners. It would be interesting to see how a mutation of this conserved Arg242_VP22_ would affect this protein *in vivo*.

The described conserved structural features and functions of VP22 and ORF52_MHV-68_ suggest that both proteins could act as protein adaptors in which different proteins are bound. Moreover, being a major tegument protein in HSV-1, VP22 has been associated with multiple protein–protein interactions, several HSV-1 protein localizations as well as protein transportation along the microtubules (Chi *et al.*, 2005; Elliott *et al.*, 1995, 2005; Elliott & O’Hare, 1998; Farnsworth *et al.*, 2007; Hafezi *et al.*, 2005; Kotsakis *et al.*, 2001; Maringer & Elliott, 2010; Maringer *et al.*, 2012; Martin *et al.*, 2002; O’Regan *et al.*, 2007a, 2010; Potel & Elliott, 2005; Stylianou *et al.*, 2009; Tanaka *et al.*, 2012; Yedowitz *et al.*, 2005). It is likely that VP22 and the structural homologue ORF52_MHV-68_ could be involved in assembling a protein scaffold consisting of other tegument proteins, thereby creating a protein bridge between the capsid and the lipid envelope. This assembly may be important for the intracellular transportation of proteins along the microtubules.

In conclusion, with a three-dimensional structure of a well-studied protein like VP22, we can now start connecting functional data with structural information. We hope that the data presented in this paper might help to spur new and directed efforts to elucidate this protein’s function. The unexpected structural similarities between VP22 and ORF52_MHV-68_ may contribute to further functional studies of other herpesviral proteins and pose intriguing questions about the evolutionary relationship of the different herpesvirus subfamilies.

## Methods

### 

#### Cloning and protein expression.

The VP22_core_ (residues 174–281) (GenBank accession number BAE87004.1) was cloned into the plasmid pNIC28-Bsa4 (GenBank accession number EF198106) containing a His_6_-tag and a TEV protease site at the N terminus. Cloning and protein expression of the VP22_core_ were performed as described previously (Hew *et al.*, 2013). The purification of VP22_core_ was performed as follows. The bacterial cells expressing VP22_core_ were pelleted and resuspended in lysis buffer (100 mM HEPES, pH 8.0, 500 mM NaCl, 10 mM imidazole pH 8.0, 10 % glycerol, 0.5 mM TCEP [Tris(2-carboxyethyl)phosphine], 0.1 mg lysozyme ml^−1^, 1 ml protease inhibitor ml^−1^ and 25 U Benzonase). Lysates were clarified and loaded on a 1 ml HisTrap HP column (GE Healthcare). The column was washed with 20 ml wash buffer (20 mM HEPES, pH 7.5, 500 mM NaCl, 10 mM imidazole, pH 7.5, 10 % glycerol and 0.5 mM TCEP) and 10 ml second wash buffer (20 mM HEPES, pH 7.5, 500 mM NaCl, 25 mM imidazole, pH 7.5, 10 % glycerol and 0.5 mM TCEP) before eluting with 5 ml elution buffer (20 mM HEPES, pH 7.5, 500 mM NaCl, 500 mM imidazole, pH 7.5, 10 % glycerol and 0.5 mM TCEP). The eluted sample was loaded on a pre-equilibrated (20 mM HEPES, pH 7.5, 300 mM NaCl, 10 % glycerol and 0.5 mM TCEP) size-exclusion column (HiLoad 16/60 Superdex 75; GE Healthcare) and eluted in 2 ml fractions. The fractions were accessed for purity on a SDS-PAGE gel and only pure fractions containing our target protein were pooled. TCEP (2 mM) was added to the pooled sample and the protein was further concentrated to 12 mg ml^−1^ before storing at −80 °C.

#### Crystallization and data collection.

Native crystals of VP22_core_ were obtained from a sitting drop experiment with drops containing 1.5 µl purified VP22_core_ protein (12 mg VP22_core_ ml^−1^) and 1.5 µl reservoir solution (40 % PEG 300 and 0.1 M phosphate citrate, pH 5) was incubated with 300 µl reservoir solution in a 24-wells sitting drop Intelli-plate (Art Robbins) at 20 °C. Native crystals were transferred to a fresh drop of reservoir solution containing 1 mM PbCl_2_ for 45 min to obtain derivative crystals. No additional cryoprotectant was added to the native and the derivative crystals before flash freezing them in liquid nitrogen.

Diffraction datasets were collected at beamline BL13C1 at the National Synchrotron Radiation Research Center (Taiwan, ROC) with the detector ADSC Quantum-315r CCD. Datasets were collected at 0.97 Å, and integrated and scaled with hkl-2000 (Otwinowski & Minor, 1997).

#### Structural determination.

The initial crystallographic model of VP22_core_ was obtained with SIRAS using AutoSol wizard and AutoBuild from the phenix suite (Adams *et al.*, 2010). The final structure was obtained after many cycles of automatic and manual structural refinement with refmac (Murshudov *et al.*, 2011) and Coot (Emsley *et al.*, 2010). The structure refinement was validated with sfcheck (Vaguine *et al.*, 1999) and the geometry of the final structure was analysed with rampage (Lovell *et al.*, 2003).

The figures of the final VP22_core_ structure were created and displayed with PyMOL (http://www.PyMOL.org/). The electrostatic potential of the solvent accessible surfaces of the protein were calculated using pdb2pqr (Dolinsky *et al.*, 2004) and the apbs plugin (Baker *et al.*, 2001) in PyMOL. The electrostatic potential contour levels were set at ±3 *kT*/*e* and the surface maps were displayed with PyMOL.

#### Structure-based sequence alignment.

The sequence alignment between VP22_core_ and ORF52_MHV-68_ was generated with a pair-wise structure-based alignment between the monomers using Chimera (Pettersen *et al.*, 2004). Sequences of the VP22_core_ and ORF52_MHV-68_ homologues from the alpha- and gammaherpesviruses were aligned by adding their amino acid sequences to the structure-based alignment. The amino acid conservation was mapped and displayed with PyMOL.

#### Multi-angle light scattering.

Light-scattering data were obtained with analytical size-exclusion chromatography (Superdex 200 5/150 GL; GE Healthcare) coupled with a multi-angle light-scattering detector (MiniDAWN TREOS; Wyatt Technology) and a refractive index detector (Optilab rEX; Wyatt Technology) on an ÄKTAmicro (GE Healthcare). An aliquot of 20 µl VP22_core_ (6 mg VP22_core_ ml^−1^) was injected onto the pre-equilibrated column (20 mM HEPES, pH 7.5, 300 mM NaCl, 10 % glycerol and 2 mM TCEP) at a flow rate of 0.3 ml min^−1^. astra 6 (Wyatt Technology) was used to determine the experimental protein molecular mass from the light-scattering data.
